# Root Distribution of Tomato Cultivated in Greenhouse under Different Ventilation and Water Conditions

**DOI:** 10.3390/plants12081625

**Published:** 2023-04-12

**Authors:** Jiankun Ge, Huanhuan Liu, Xuewen Gong, Zihui Yu, Lusheng Li, Yanbin Li

**Affiliations:** Henan Key Laboratory of Crop Water Use, School of Water Conservancy, North China University of Water Resources and Electric Power, Zhengzhou 450046, China

**Keywords:** greenhouse tomato, irrigation, NRLD distribution model, yield, ventilation

## Abstract

Mastering root distribution is essential for optimizing the root zone environment and for improving water use efficiency, especially for crops cultivated in greenhouses. Here, we set up two irrigation amount levels based on measurements of the cumulative 20 cm pan evaporation (*E_p_*) (i.e., *K*_0.9_: 0.9 *E_p_*; *K*_0.5_: 0.5 *E_p_*), and three ventilation modes through opening the greenhouse vents at different locations (*T_R_*: open the roof vents only; *T_RS_*: open both the roof and south vents; *T_S_*: open the south vents only) to reveal the effects of the ventilation mode and irrigation amount on the root distribution of greenhouse tomato. Six treatments were designed in blocks with the ventilation mode as the main treatment and the irrigation amount as the vice treatment. On this basis, the normalized root length density (NRLD) model of six treatments was developed by considering air environment, soil water and temperature conditions, root length density (RLD) and yield. The results showed that air speed of the *T_RS_* was significantly higher than *T_R_* and *T_S_* (*p* < 0.01), and the air temperature and relative humidity under different ventilation showed the rule: T*_R_* > *T_S_* > *T_RS_*. There was a significant third-order polynomial function relationship between NRLD and soil depth, and the coefficient of the cubic term (*R*_0_) had a bivariate quadratic polynomial function relationship with irrigation amount and air speed (determination coefficient, *R*^2^ = 0.86). Root mean square errors of the simulated and measured value of NRLD under *T_R_*, *T_RS_* and *T_S_* were 0.20, 0.23 and 0.27 in 2020, and 0.31, 0.23 and 0.28 in 2021, respectively, normalized root mean squared errors were 15%, 17%, 20% in 2020, and 23%, 18% and 21% in 2021. The RLD distribution ratio from the ground surface to a one-quarter relative root depth was 74.1%, and 88.0% from the surface to a one-half relative root depth. The results of the yield showed that a better combination of ventilation and irrigation was recommended as *T_RS_* combined with *K*_0.9_.

## 1. Introduction

With the rapid development of modern agriculture, the cultivation technology of solar greenhouses was used to improve vegetable yield, quality and water use efficiency, and had gradually attracted much attention from farmers, especially in northern China [1]. Many greenhouses have a semi-enclosed structure, in which crops are greatly influenced by meteorological factors and the soil environment [2,3]. The root system is an important organ for the transfer of soil moisture and crop water; mastering the root distribution characteristics and water absorption law can well explain the response mechanism of crops to water and environmental stress.

Crop root distribution characteristics in greenhouses have been reported in some previous studies. For example, the root distribution characteristics of pepper and tomato grown in greenhouse were studied by Zapata-Sierra et al. (2021) [4], who indicated that 90% of the root system was concentrated in the range of 0–9 cm (pepper) and 0–11 cm (tomato). Different irrigation amounts would lead to different water conditions around the roots [5,6], thus affecting the growth and distribution of crop roots. Insufficient irrigation would inhibit tomato root growth, while excessive irrigation may lead to a waste of water resources [7,8]. Wu et al. (1999) [9] studied various crop root distribution characteristics and found that the relative root length density and relative soil depth can be described as probabilistic functions. Zuo et al. (2006) [10] built a relative root length density distribution model for winter wheat by using the normalization method. Thereafter, Ning et al. (2019) [11] compared the normalized model with the exponential distribution model, linear distribution model and piecewise distribution model, and indicated that the root length density calculated by the normalized model was in good agreement with the measurements, with an error of 4.0%. Some studies have also reported that the normalized method can better express the spatial distribution characteristics of roots, and calculate root length density based on these theories. However, these studies mainly focus on crops grown under field conditions, and few reports focus on vegetables grown in greenhouse conditions.

In recent years, some studies have tried to analyze the root distribution under different ventilation modes, and have concluded that ventilation is also one of the important factors affecting root growth in greenhouse conditions. It is known that crop roots need soil oxygen to maintain respiration and growth properly [12] and that most oxygen (O_2_) is obtained directly through diffusive gas exchange from the atmosphere to the inter-root soil. Ventilation can increase the concentration of oxygen in greenhouse. Ventilation can also reduce the physiological stress of the crop by changing the temperature and humidity in the greenhouse [13]. At the same time, suitable temperature and humidity would reduce the production of crop pests and diseases [14], hence promoting crop growth. In addition, the effect of different drip irrigation amounts on tomato root distribution was also studied [15,16], and the results indicated that mild water stress favored the growth of deep root. Ullah et al. (2021) [17] reported that root length and root surface area can significantly increase by reducing the irrigation amount. Mild water stress could promote root growth and plant development, which is beneficial for improving water use efficiency [18].

One study found that ventilation and irrigation affected crop root growth and distribution by altering the greenhouse environment and soil structure, and suitable ventilation mode promoted root growth [19], while another study found that excessive irrigation led to poor root zone permeability, thus limiting root development [20]. In addition, crop root growth indicators, such as root length, root diameter, root surface area and root volume, also changed with ventilation and irrigation management. Previous studies on root systems mainly focused on the distribution patterns of crop root length density in the profile [21]. Root length density represents the total length of the root system per unit volume of soil and reflects the number of roots. Relative root length density represents the proportion of root length distributed at different relative depths and it is used to describe the relative distribution of root length density. Novak (1994) [22] built an exponential distribution model of root length density by analyzing the horizontal and vertical distribution of the underground root system. Yang et al. (2009) [23] established the relative root length density distribution model of wheat by polynomial fitting and verified it through measured and simulated water content with high accuracy. Zuo et al. (2004) [24] established a nonlinear equation with four parameters to simulate the root distribution. Wu et al. (1999) [9] expressed the plant root distribution in terms of a third-order polynomial equation. However, these models only described the distribution of root systems in the horizontal and vertical directions [25,26] and had not considered the test conditions. Therefore, test variables were added to the simulation of root distribution to obtain a more accurate model of root distribution.

From the above research results, we know that ventilation and irrigation amount affect root growth simultaneously in greenhouse conditions. Crop root growth and distribution also affect fruit yield and water use efficiency [27]. However, few studies have focused on the interaction effect of ventilation and irrigation on root distribution, and root length density models under interactive conditions are also lacking. Therefore, two years of studies were conducted in a solar greenhouse to achieve the following objectives: (1) analyze the variations in soil water content, temperature and meteorological factors under different ventilation and irrigation amounts; (2) explore the root distribution and root length density of tomato with drip irrigation under different combinations; (3) establish the relative root length density distribution model and evaluate its performance; and (4) explore the effect of an optimal combination of ventilation and water conditions on tomato yield.

## 2. Results

### 2.1. Meteorological, Soil Water and Temperature Condition

#### 2.1.1. Variations in Meteorological Factors

**Figure 1:**
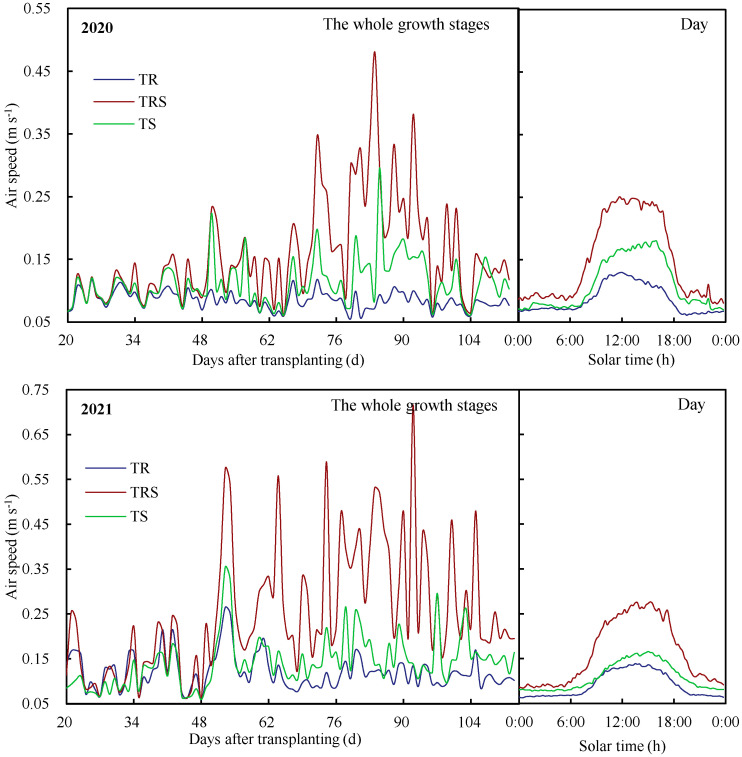
Variations in air speed inside greenhouses under different ventilation treatments in 2020 and 2021.

Variations in air speed inside the greenhouse are shown in Figure 1; the right panel indicates the daily average air speed during the whole growth stages, the left panel indicates the variation in air speed with the number of days after transplanting. We found that the magnitude of air speed was *T_RS_* > *T_S_* > *T_R_*, and it varied between 0.06 and 0.27 m s^−1^ in the *T_R_* treatment, 0.06 and 0.72 m s^−1^ in the *T_RS_* treatment, and 0.06 and 0.36 m s^−1^ in the *T_S_* treatment in the two study years. The difference in air speed for the three ventilation treatments began to increase significantly 60 days after transplanting, and also significant differences between *T_RS_* and the other two treatments were found in our study (Table 1), which was entirely due to the different opening states of these vents.

**Figure 2:**
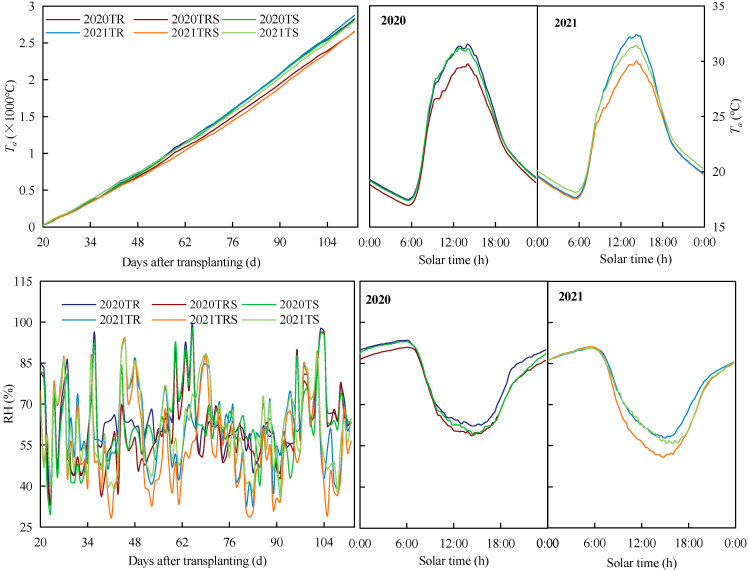
Characteristics of air temperature (*T_a_*) and relative humidity (RH) under different ventilation treatments in 2020 and 2021.

Variations in *T_a_* and RH under the three ventilation treatments are shown in Figure 2; the right and middle panels indicate the average *T_a_* and RH during the whole growth stages, and the left panel indicates the cumulative *T_a_* and RH with the number of days after transplanting, for the whole growth stages, and the opposite trends for daily *T_a_* and RH are obvious [28]. The average daily *T_a_* and RH of the *T_RS_* treatment were highly significantly different from the *T_R_* and *T_S_* treatments (*p* < 0.01) in the middle of the day during the ventilation period (9:00–17:00) for the two seasons. The daily *T_a_* and RH of the *T_RS_* were 28.06 °C and 59.36%, which were 6.4%, 5.1% and 7.4%, 5.5% lower than the *T_R_* and *T_S_*, respectively. The cumulative temperature was closer between *T_R_* and *T_S_* but ~6.48% and ~5.38% higher than *T_RS_* in 2020 and 2021, respectively. The daily *T_a_* and RH of *T_RS_* were 28.06 °C and 59.36%, which were 6.4% and 5.1% and 7.4% and 5.5% lower than those of *T_R_* and *T_S_*, respectively. The maximum RH (96.61%) during both growth periods occurred in *T_R_*, which was 1.07% and 1.12% higher than that *T_RS_* and *T_S_*, respectively.

#### 2.1.2. Variations in Soil Water and Temperature

**Figure 3:**
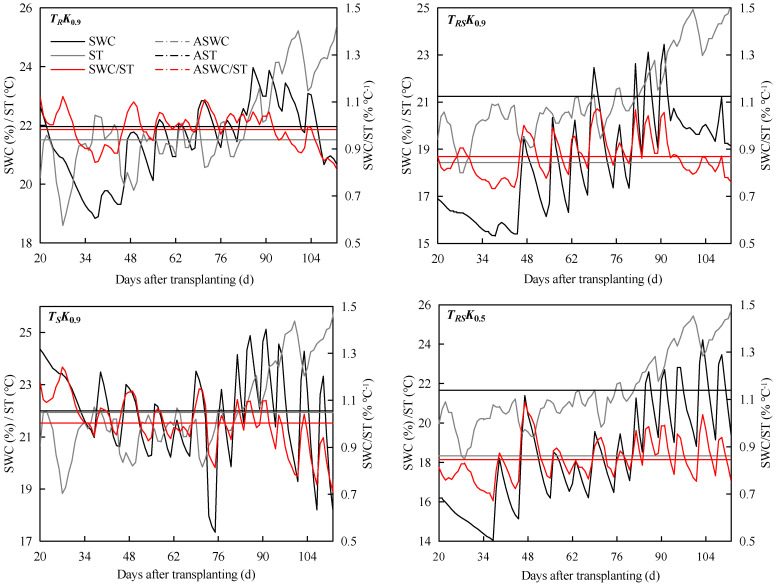
Change curves of soil temperature (ST), soil water content (SWC) and ratio of soil water content to temperature (SWC/ST) in 2021. ASWC is the average soil water content, AST is the average soil temperature in the 0–20 cm soil layer and ASWC/ST is the average soil water-heat ratio.

Crop root development was largely affected by soil water and heat conditions [29,30]. Soil temperature (ST), soil water content (SWC) and the ratio of water to heat ratio (SWC/ST) are shown in Figure 3, where SWC/ST was calculated by the ratio of the average volumetric water content to soil temperature within the 0–20 cm soil layer. A general opposite trend between ST and SWC was observed during the whole growth stage. The average SWC/ST of *T_RS_* was 0.87% °C^−1^ in *K*_0.9_, which was 11.2% and 13.3% lower than *T_R_* and *T_S_*, respectively. However, for *T_RS_*, the ST and SWC of the two irrigation treatments (*K*_0.9_ and *K*_0.5_) were significantly different, with an overall higher soil temperature and water content in *K*_0.9_ treatment than *K*_0.5_ treatment, while the difference of SWC/ST between *K*_0.5_ and *K*_0.9_ was not significant.

### 2.2. Distribution of Root Length Density

**Figure 4:**
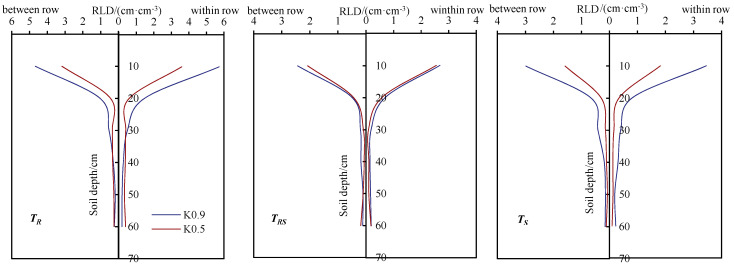
Tomato RLD distribution under different ventilation and irrigation amounts in 2021.

The distribution of RLD was directly affected by the soil environment. The tomato RLD distribution of each treatment is shown in Figure 4. RLD under the same ventilation treatment showed that *K*_0.9_ > *K*_0.5_, and RLD within rows was higher than between rows. *K*_0.9_ and *K*_0.5_ within rows increased by 19.9% and 18.5% (*T_R_*), 15.0% and 21.3% (*T_RS_*) and 19.0% and 14.9% (*T_S_*) compared to between rows. The RLD under *T_R_* and *T_S_* varied from 10.44 to 15.20 cm cm^−3^ and 5.00 to 9.94 cm cm^−3^, respectively, while *T_RS_* was between 6.55 and 7.58 cm cm^−3^ under the *K*_0.9_ and *K*_0.5_ treatments, respectively. There was a larger change between *T_R_* and *T_S_*, while the RLD of *T_RS_* was similar under two different irrigation treatments. A similar phenomena can be found where the RLD under the three ventilation treatments decreased gradually with the deepening of the soil layer below 10 cm soil depth.

### 2.3. Development of the NRLD Model

Equation (7) was used to fit the relationship between relative RLD and soil depth under different treatments in 2021, resulting in correlation coefficients shown in Table 2. *R*^2^ was greater than 0.96 for all treatments, and the *F*−statistics reached a highly significant level (*p* < 0.01). There was a highly significant correlation between soil depth and RLD, indicating that the function was good. Correlation coefficients for each treatment (*R*_0_, *R*_1_ and *R*_2_) can represent the trend of curve and varied from −97.16 to −79.01, 128.15 to 152.77 and −72.73 to −66.67, respectively. Under the same ventilation mode, *R*_0_ and *R*_2_ decreased and then increased, while *R*_1_ increased then decreased with the increase in irrigation amount. Under the same irrigation amount, *R*_0_ and *R*_2_ increased then decreased, while *R*_1_ decreased then increased with the increase in air speed. *R*_3_ represented the relative RLD value at the relative depth Z_r_ = 0. As no root system existed at soil surface (0 cm), it could be regarded as a theoretical value and did not have practical significance.

**Figure 5:**
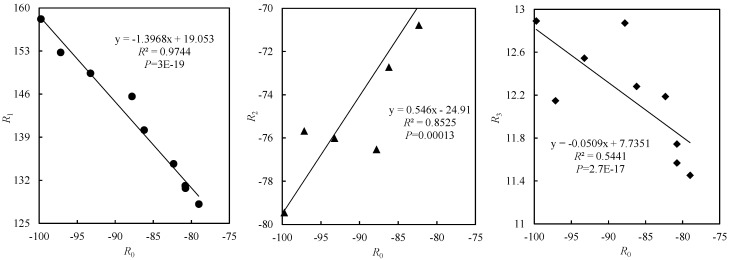
Relationship between parameters *R*_1_, *R*_2_, *R*_3_ and *R*_0_. Relationship between parameters *R*_1_ and *R*_0_, relationship between parameters *R*_2_ and *R*_0_ and relationship between parameters *R*_3_ and *R*_0_.

The ventilation effect from 10:00 to 16:00 was used as the research object. A generalized NRLD distribution model was established according to the irrigation amount (*K*) and air speed (*S*), and then the related parameters were obtained. We found a highly significant correlation between *R*_1_, *R*_2_, *R*_3_ and *R*_0_ (*p* < 0.01) (Figure 5). With *K* and *S* as independent variables and parameter *R*_0_ as the dependent variable over the whole growth stages, the binary quadratic polynomial function could be fitted by using the *Levenberg–Marquardt* method combined with the general global optimization algorithm by using 1stOpt software (*R*^2^ of 0.86), which could be derived under different irrigation amounts and air speeds in the equation for parameter *R*_0_.

(1) $$R_{0}=-174.502-1.343K+30.188S+3.411\;\times \;10^{-3}K^{2}-0.924S^{2}-0.00717\mathrm{KS}$$

where *K* is the irrigation amount, mm; and *S* is the average air speed over the whole growth stage, m s^−1^.

Based on the relationship equation between parameters *R*_1_, *R*_2_, *R*_3_ and *R*_0_, and the calculated Equation (1), expressions of the one-dimensional NRLD distribution model for drip-irrigated greenhouse tomatoes were obtained as follows:

(2) $$\left\{\begin{array}{c} NRLD=R_{0}Z_{r}^{3}+\left(-1.3968R_{0}+19.053\right)Z_{r}^{2}+\left(0.546R_{0}-24.91\right)Z_{r}-0.0509R_{0}+7.7351\\ R_{0}=-174.502-1.343K+30.188S+3.411\times 10^{-3}K^{2}-0.924S^{2}-0.00717KS \end{array}\right.$$

### 2.4. Validation and Application of the NRLD Distribution Model

#### 2.4.1. Validation

**Figure 6:**
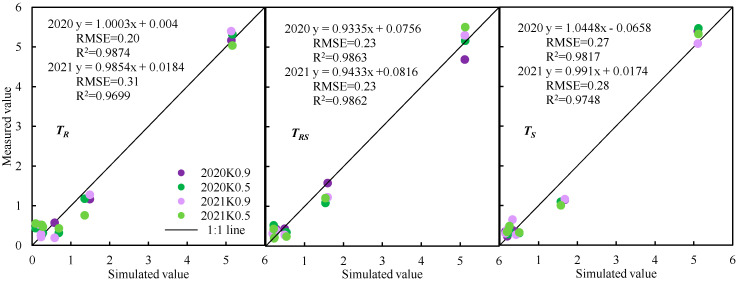
Comparison of simulated and measured NRLD values for greenhouse tomato under different ventilation and irrigation amounts.

The NRLD distribution model was validated by using measured data in 2020 and 2021. The simulated values of relative RLD at each relative depth (Z_r_) were obtained by substituting the irrigation amount and average air speed into Equation (2). The measured and simulated values were compared to obtain a 1:1 histogram at three ventilation treatments (data points = 72) (Figure 6) and a plot of NRMSE statistics (data points = 12) (Table 3). Under different irrigation amounts, the simulated values of three ventilation treatments agreed well with the measured values, and the RMSE under *T_R_*, *T_RS_* and *T_S_* was 0.20, 0.23 and 0.27 in 2020, and 0.31, 0.23 and 0.28 in 2021, respectively. Table 3 shows the NRMSE between the simulated and measured values of the relative RLD in the two study years. The NRMSE between the simulated and measured values under *T_R_*, *T_RS_* and *T_S_* was 19%, 18% and 21%, respectively, indicating that the performance of the model was perfect.

#### 2.4.2. Application

The relative RLD can be simulated by using Equation (2) when the maximum root depth (Z_max_) and RLD are known. A relative RLD distribution model was used to accurately estimate the ratio of root length to total root length (RL/TRL) under different relative sampling depths by using two irrigation amounts and three ventilation treatments in 2020 (irrigation amount: *K*_0.9_ = 247.5 mm, *K*_0.5_ = 137.5 mm; average air speed: *T_R_* = 0.092 m s^−1^, *T_RS_* = 0.152 m s^−1^, *T_S_* = 0.115 m s^−1^). The relative RLD and RL/TRL were estimated and compared with the measured data (Table 4). The drip-irrigated tomato roots were mainly distributed in the upper soil layer (0–20 cm), which was similar to the research results of Li et al. (2020) [31]. Root length from the surface to a one-quarter depth of relative root system accounted for 74.1% of total root length, while that from the surface to a one-half depth of relative root system accounted for 88.0%. Under the same irrigation amount, RL/TRL increased then decreased with the increase in air speed. Under the same ventilation, RL/TRL increased gradually with the increase in irrigation amount, the difference between the simulated and measured values was greater in the high irrigation amount than in the low irrigation amount, and measured values were lower than the simulated values. Using 2020 as an example, the root distribution under different ventilation and irrigation amount with pan evaporation coefficients of 0.6, 0.8 and 1.0 was predicted (Table 5). The one-dimensional NRLD distribution model can clarify the proportion of root distribution at different soil layers under different ventilation and irrigation amounts, which is beneficial to guide the middle and late ventilation and irrigation management of greenhouse tomatoes.

### 2.5. Root System and Yield

The yield and RLD for different treatments in 2020 and 2021 are shown in Table 6. The *T_RS_K*_0.9_ treatment has the highest yield (147.6 t ha^−1^ and 148.1 t ha^−1^), followed by the *T_S_K*_0.9_ treatment (143.1 t ha^−1^ and 144.5 t ha^−1^). The lowest is the *T_RS_K*_0.5_ treatment (119.4 t ha^−1^ and 126.3 t ha^−1^). At the highest yield, the root length density is not the highest, but it is at the middle level of the six treatments. Considering the tomato yield, root length density, irrigation amount and ventilation, the combination of *T_RS_* and *K*_0.9_ is beneficial to increase tomato yield.

## 3. Discussion

### 3.1. Effect of Ventilation Mode and Irrigation Amount on Greenhouse Environment

The greenhouse environment was significantly affected by the ventilation mode and irrigation amount [32,33,34]. There was a highly significant difference (*p* < 0.01) in air speed under different ventilation conditions. In our study, the air speed under *T_RS_* was significantly higher than *T_R_* and *T_S_*. The magnitude of the indoor air speed was a good measure of the water vapor diffusion capacity of the greenhouse [35]. *T_a_* and RH were also influenced by the ventilation mode, which showed that *T_R_* > *T_S_* > *T_RS_* in our study. The air speed regulated the *T_a_* and RH around the leaves, as well as the rate of water vapor diffusion, which had a significant impact on the internal environment of the greenhouse [36]. For instance, *T_a_* and RH of *T_RS_* were significantly lower than *T_R_* (Table 1). ST and SWC, as important indicators of the soil environment, were influenced by the irrigation amount. The ASWC/ST in the 0–20 cm soil layer of *T_R_* was significantly higher than *T_RS_*, because air speed had a greater effect on crop evapotranspiration and low air speed slowed the soil water depletion process.

### 3.2. Effect of Ventilation Mode and Irrigation Amount on RLD Distribution

The tomato roots were dominantly concentrated in shallow soils (0–20 cm) in the vertical direction, which is the same as Liu et al. [37], who indicated that the tomato root system had an initial growth at a faster rate than the infiltration stress at the soil surface, and the root system extended deeper and further, with a maximum root length density in the range of 10–20 cm. However, Zapata-Sierra et al. (2021) [4] showed that tomato roots were mainly distributed in the 0–11 cm or 15.5 cm soil layer, which may be due to different sampling accuracies. Most studies have found that the maximum root depth was in the range of 60–80 cm [8,38], which was similar to the present study, i.e., the maximum rooting depth of greenhouse tomatoes could be up to 80 cm [39]. Wang et al. (2021) [40] showed that for drip-irrigated tomatoes, the RLD decreased with decreasing irrigation, and the RLD under the same ventilation in this study was *K*_0.9_ > *K*_0.5_. In this study, root length increased then decreased from the surface to one-half of the relative root depth, with 85.8%, 88.5% and 89.7% for *T_R_*, *T_RS_* and *T_S_*, respectively, indicating that excessive air speed did not increase root amount at one-half, and suitable ventilation is conducive to the growth of tomato roots. Similar findings have been proven by Ge et al. (2019) [41]. The reason was primarily due to excessive air speed causing the crop stomata to close, affecting transpiration and preventing the roots from absorbing water and nutrients properly. A higher proportion of deep soil root distribution in better plants would be able to absorb nutrients from deep soil to improve yield [42,43]. Shu et al. (2020) [44] analyzed the effect of different drip irrigation rates on tomato roots and found that moderate deficit irrigation was beneficial to root “active rooting”, which promoted the development of aboveground parts by absorbing water and nutrients from soil, increasing yields and harvests and improving water use efficiency.

### 3.3. Performance of NRLD Distribution Models in Different Ventilation Mode and Irrigation Amount

The NRLD of greenhouse tomato under different ventilation and irrigation amounts was constructed in this study, and the model parameters *R*_0_, *R*_1_ and *R*_2_ can reflect the simulation accuracy. *R*_3_ is a theoretical value of the NRLD at the surface, and other parameters accurately describe the accuracy of root development fit in soil. Wu et al. (1999) [9] used the normalization method to establish a third-order polynomial function for the relative RLD of wheat and maize, and the function was able to accurately simulate root variation in different soil layers, which was similar to our study. Use of normalization had a good applicability for root modeling. Compared with the maize root system model established by Zou et al. (2018) [28], the parameters *R*_0_ and *R*_2_ fitted in this study were small, while the parameters *R*_1_ and *R*_3_ were large. The mean value of *R*_3_ was 12.19, which was higher than the value of 5.51 fitted by Zou et al. (2018) [28]. The reason for the difference were that the root system data collected by Zou et al. were all roots, while the tomato root system in our study was partial roots, resulting in the differences in model parameters. However, the *R*^2^ of the binary quadratic polynomial fit (Equation (1)) under different ventilation modes and irrigation amounts was 0.86, higher than the fitting accuracy of Zou et al. (*R*^2^ of 0.84), which may be caused by differences in crop type, root sampling depths and sample numbers.

### 3.4. Application of the NRLD Distribution Model

The RLD distribution characteristics of greenhouse tomatoes and the estimated proportion of root distribution in different soil layers were obtained by using the NRLD distribution model, quantifying the influence of ventilation and irrigation on root growth. Therefore, we provide a theoretical basis for the study of root growth models, soil water and solute transport. Previous studies indicated that relative RLD distribution models were better applied to other models for simulating soil water transport and other processes to elucidate crop water uptake patterns. For example, Ning et al. (2015) [45] developed an NRLD distribution model for sunflower to validate and estimate the RLD distribution, which was applied to the Hydrus model to simulate water transport with good results and improve the accuracy of soil water and salt transport simulation. Zuo et al. (2004) [24] developed a model for the NRLD distribution of wheat and applied the measured water content profile to estimate the relative RLD distribution, which provided a basis for the continuous simulation of soil water transport. Compared to the traditional method of obtaining RLD, this model only needs the maximum rooting depth and average RLD at any soil layer. Using the NRLD distribution model, RLD data could be obtained, and real-time information on the proportion of roots in different soil layers is important for accurate ventilation and irrigation management. In this study, NRLD distribution models were established using different ventilation and water treatments to estimate the proportion of root distribution under different treatments. The results showed that the proportion of root length in the upper half of the root zone for *K*_0.9_ and *K*_0.5_ averaged 88.9% and 87.1%, respectively, and the simulated values were close to the measured values. Combining the 2020 ventilation data and setting evaporation pan coefficients of 1.0, 0.8 and 0.6 for these three irrigation treatments, the results show that reducing irrigation leads to an increase in the proportion of deep roots, and conversely, an increase in the proportion of shallow roots. Combined with the NRLD distribution model, the proportion of greenhouse drip-irrigated tomato roots distributed in different soil layers could be determined in real time, providing a reference for optimizing water and environmental management strategies for crops in middle and late periods. For instance, when the proportion of shallow soil roots is high, the irrigation amount could be reduced, and ventilation could be controlled in an appropriate amount to save water and increase yields.

### 3.5. Effect of Ventilation and Irrigation Amount on Tomato Yield

Ventilation changes the micro-environment in which crops grow, and also affects the temperature and humidity of the air layer and soil layer, and micro-environment varies depending on the location and size of the vents in the greenhouse (Figure 1, Figure 2 and Figure 3). The differences in tomato yields under the three ventilation treatments are not significant (Table 6), which could be due to the high temperature in the greenhouse affecting colored fruits of the crops, and did not have an effect on crop yield [46]. Irrigation treatments had a significant effect on the yield for the greenhouse tomatoes. Under the same ventilation treatment, the yield for the high irrigation treatment (*K*_0.9_) was significantly higher than the low irrigation treatment (*K*_0.5_), which is the same as the results of Li et al. (2020) [47], in which a water deficit would be caused under low irrigation treatment, leading to difficulties in water uptake by plant roots, limiting nutrient translocation, affecting carbohydrate synthesis and fruit quantity and quality, as well as affecting plant physiology and reducing plant yield. In addition, a low irrigation amount would lead to a reduced stomatal closure and photosynthetic capacity of plants, and then result in a reduced substance synthesis and lower yields [48]. In our study, a combination of *T_RS_* and *K*_0.9_ treatments was the most beneficial for greenhouse tomato yield.

## 4. Materials and Methods

### 4.1. Experimental Site and Design

The experiment was conducted from March to July in 2020 and 2021 in a solar greenhouse at the Xinxiang Integrated Experimental Base of the Chinese Academy of Agricultural Sciences (35°9′ N, 113°5′ E, 78.7 m above sea level). The greenhouse walls are made of brick and concrete construction, with an area of 510 m^2^. The direction of the greenhouse is east–west, and it sinks 0.5 m. A steel frame construction is used to support the roof of the greenhouse and is covered with a 0.2 mm thick polyethylene drip-free film to maintain the air temperature inside. Meanwhile, 5 cm thick insulation quilts are used to maintain warmth at the seedling stage. There are three vents, one on the roof (60 m × 30 cm) and another on the bottom of the south side (60 m × 1.5 m). The soil in the greenhouse at a depth of 0–100 cm is a silt loam, including 16.3% clay, 77.1% silt and 6.6% sand. Mean field water capacity and wilting water content at a soil depth of 0–100 cm are 0.31 and 0.11 cm^3^ cm^−3^, respectively, with an average bulk density of 1.59 g cm^−3^.

Tomato seedlings “*Solanum lycopersicum* L. *c.v*. Jinpeng M6” were transplanted on 4 March 2020 and 7 March 2021. The size of the experimental plot was 8.0 m long and 2.2 m wide, and a wide (65 cm) and narrow (45 cm) row planting mode was used, with an interval of 30 cm. The planting density was 5.7 trees m^−2^. Drip irrigation was used, with a drip head flow rate of 1.1 L h^−1^. Each plot was replicated three times, and six treatments were designed in blocks with the ventilation mode as the main treatment and the irrigation amount as the vice treatment. Here, three ventilation treatments were set: *T_R_* (open the roof vents only), *T_RS_* (open both the roof and south vents), *T_S_* (open the south vents only), and two irrigation treatments were set according to the cumulative water evaporation (*E_p_*) from a standard 20 cm evaporation pan (20 cm diameter and 11 cm deep): *K*_0.9_ (0.9 *E_p_*) and *K*_0.5_ (0.5 *E_p_*). The irrigation events were performed based on the average surface evaporation of the three ventilation treatments. According to the results of our previous research [49], soil water content of 0–60 cm accounts for 80–90% and 60–65% of field water capacity, respectively, under an irrigation amount of 0.9 *E_p_* and 0.5 *E_p_*. The evaporation pan was placed 30 cm above the crop canopy and adjusted according to tomato plants’ development. The evaporation water amount was measured every day at 8:00 using a measuring cylinder with an accuracy of 0.1 mm, and 20 mm of distilled water was refilled after the measurement. When the accumulated water evaporation reaches 20 ± 2 mm, irrigation events was conducted [50]. The irrigation amount (*I_r_*) was calculated according to Equation (3).

(3) $$I_{r}=E_{p}\times \varphi$$

where *I_r_* is the irrigation amount, mm; *E_p_* is the accumulated evaporation, mm; and *φ* is the water surface evaporation coefficient.

A water meter with an accuracy of 0.001 m^3^ was installed at the head of each plot to precisely control the irrigation amount. A supplementary irrigation amount of 20 mm was performed by drip irrigation after transplanting to maintain the seedlings alive. In this study, 112 kg hm^−2^ urea (containing 46% N), 150 kg hm^−2^ potassium sulfate (containing 50% K_2_O), and 120 kg hm^−2^ superphosphate (containing 14% P_2_O_5_) were used as base fertilizers and ploughed to a depth of 16 cm with a rotary spade. Thereafter, differential pressure fertilizer tanks were used for topdressing urea at 18.8 kg hm^−2^ and potassium sulfate at 25 kg hm^−2^. The fertilizer was applied four times when the first, second, third and fourth truss fruits began to expand. The agronomic practices (e.g., topping, spraying, fruit thinning) were the same as those used locally. The irrigation amounts were 247.5 and 245.7 mm (*K*_0.9_) and 137.5 and 136.5 mm (*K*_0.5_) in 2020 and 2021, respectively.

### 4.2. Measurement

#### 4.2.1. Environmental Factors

Air speed in the greenhouse was monitored by using an anemometer (Wind Sonic, Gill, UK) at the vents with an accuracy of ±0.02 m s^−1^. Data were collected every 5 s, and the 15 min average was recorded in a CR1000 data logger (Campbell Scientific Inc., Logan, UT, USA). The air temperature and relative humidity were measured by using an automatic climate station (CS215, Campbell Scientific, Inc, Monterrey, CA, USA) with accuracies of 0.02 °C and 0.18 °C, and 15 min averages were calculated and stored.

#### 4.2.2. Soil Water Content

A ZL6 cloud data collector (METER Group, USA) with an accuracy of 1 × 10^−6^% was used to determine the water content of the soil layer at 0, 10, 20, 30, 40 and 60 cm in the middle of two drip heads of the same drip tape [51], with data collected automatically every 15 min.

#### 4.2.3. Soil Temperature

An eight-channel soil temperature logger (JL-04, Ningbo, China) with an accuracy of 0.1 °C was used to monitor soil temperatures at 0, 10, 20, 30, 40 and 60 cm, with data collected automatically at 30 min intervals.

#### 4.2.4. Root Distribution

**Figure 7:**
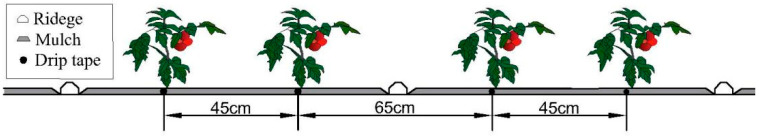
Diagram of the greenhouse tomato growing pattern.

At the end of picking, root systems were removed from the soil depth between 0 and 60 cm at different orientations by using a layered segmental soil auger with an auger diameter of 7 cm. The soil enclosing the roots was removed at intervals of 10 cm within and between rows (Figure 7). Tomato roots were placed in mesh bags, rinsed and scanned into JPG files using a 4800 (H) × 9600 (V) dpi (MRS-9600TFU2L, WANSHEN, China) scanner and analyzed for morphological characteristics, root length and surface area image were analyzed by software (Win RHIZO Pro2004 b, Canada). Root length density (RLD, cm cm^−3^) was calculated according to Equation (4):

(4) RLD=RL/V

where RLD is the root length density, cm cm^−3^; RL is the root length in different soil layers, cm and V is the root auger volume (384.85 cm^3^).

#### 4.2.5. Yield

Twenty plants were selected in the middle of each plot to measure yield, and this was repeated 3 times. When the tomato fruits were picked, an electronic balance with an accuracy of 0.005 kg was used to weigh the tomato and record the number to calculate tomato yield.

### 4.3. Root Length Density Distribution Model

To facilitate the modeling of tomato root length density under different treatments, the normalization method [9] was used to convert the penetration depth of tomato roots at different soil layers to a standardized root depth in the range of 0 to 1. The root length density was expressed as a generalization of the relative root length density by using the following equation:

(5) $$Z_{r}=Z_{i}/Z_{max}$$

(6) $$NRLD(Z_{r})=\frac{RLD(Z_{r})}{\int_{0}^{1}RLD(Z_{r})dZ_{r}}$$

where Z_r_ is the standardized root depth, between 0 and 1, dimensionless; Z_i_ is the depth of the rooted soil layer, cm; Z_max_ is the maximum rooting depth, cm, obtained for the root-free soil layer, and the maximum rooting depth in this experiment is 80 cm; NRLD (Z_r_) is the relative root length density value, dimensionless; and RLD (Z_r_) is the root length density value at Z_r_, cm cm^−3^.

A third-order polynomial was used to fit the mean values of NRLD (Z_r_) at each lateral position at the relative sampling depth (Z_r_) based on previous studies [9,52]. The equation is as follows:

(7) $$\mathrm{NRLD}(Z_{r})=R_{0}Z_{r}^{3}+R_{1}Z_{r}^{2}+R_{2}Z_{r}+R_{3}$$

where *R*_0_, *R*_1_ and *R*_2_ are polynomial parameters and *R*_3_ represents the theoretical value of the NRLD at the surface.

### 4.4. Model Evaluation

The regression fitting method was used to model the relative root length density distribution for the 2021 root data, and the model was validated using the 2020 measured data. Microsoft Excel 2010 and SPSS 26.0 were used for data processing and graphing. Model simulations were carried out using 1stOpt software, and the model was evaluated using the coefficient of determination (*R*^2^), root mean square error (RMSE) and normalized root mean squared error (NRMSE).

(8) $$R^{2}=\frac{{\left\{\sum_{i=1}^{N}\left[\left(Y_{i}-\bar{Y}\right)\left({\hat{Y}}_{i}-\bar{\hat{Y}}\right)\right]\right\}}^{2}}{\sum_{i=1}^{N}\left[{\left(Y_{i}-\bar{Y}\right)}^{2}\right]\sum_{i=1}^{N}\left[{\left({\hat{Y}}_{i}-\bar{\hat{Y}}\right)}^{2}\right]}$$

(9) $$\mathrm{RMSE}={\left[\sum_{i=1}^{N}{\left(Y_{i}-{\hat{Y}}_{i}\right)}^{2}/N\right]}^{\frac{1}{2}}$$

(10) $$\mathrm{NRMSE}=\frac{\mathrm{RMSE}}{\overset{-}{Y}}$$

where N is the number of samples; Y_i_ is the measured values; ${\hat{Y}}_{i}$ is the simulated values; $\bar{Y}$ is the measured mean value; and $\bar{\hat{Y}}$ is the simulated mean value. *R*^2^ is close to 1, the better the correlation is; RMSE can indicate the average difference between the simulated and observed values, and the closer it is to 0, the smaller the deviation is. NRMSE indicates how good the model simulation performance is; when NRMSE < 10%, the model simulation performance is excellent; when 10% ≤ NRMSE < 20%, the model simulation performance is good; when 20% ≤ NRMSE < 30%, the model simulation performance is average; and when NRMSE ≥ 30%, the simulation performance is considered poor.

## 5. Conclusions

In this study, we investigated the effects of different ventilation and irrigation amounts on air environment, soil water and temperature conditions and the root distribution of greenhouse tomatoes under drip irrigation. The main conclusions were as follows.

The ventilation and irrigation amount had a significant effect on soil water, temperature and meteorological factors. Ventilation mainly had a significant effect on meteorological factors. *T_RS_* was more beneficial to greenhouse gas exchange than *T_R_* and *T_S_*. *T_a_* and RH were affected by the ventilation treatments in the following manner: *T_R_* > *T_S_* > *T_RS_*. The ventilation and irrigation amount had an interaction effect on soil water and temperature.

The root distribution differences were substantially influenced by both the irrigation amount and ventilation. However, consistent trends of decreasing root length density with deepening of the soil layer were observed below the 10 cm level. Thereafter, a relative root length density distribution model was established according to the following principles: firstly, the relationship between NRLD and relative depth of soil profile was a third-order polynomial; secondly, the coefficient of cubic term (*R*_0_) had a bivariate quadratic polynomial relationship with the irrigation amount and air speed. The RMSE between the simulated and measured values of NRLD at *K*_0.9_ and *K*_0.5_ were 0.20, 0.23 and 0.27 in 2020 and 0.31, 0.23 and 0.28 in 2021, respectively, indicating that performance of the model was perfect.

The irrigation amount and ventilation had significant effects on RLD and yield. The combined treatment of *T_RS_* and *K*_0.9_ was the most beneficial to increase tomato yield.

## Figures and Tables

**Table 1:** Air speed (m s^−1^), air temperature (*T_a_*, °C) and relative humidity (RH, %) inside the greenhouse under different ventilation treatments in 2020 and 2021.

Year	Treatment	Cropping Seasons			Time (9:00–17:00)		
		*T_R_*	*T_RS_*	*T_S_*	*T_R_*	*T_RS_*	*T_S_*
2020	Air speed	0.085 ± 0.00 c	0.159 ± 0.01 a	0.113 ± 0.01 b	0.086 ± 0.00 c	0.148 ± 0.01 a	0.110 ± 0.01 b
	*T_a_*	30.40 ± 0.62 a	28.55 ± 0.54 b	30.22 ± 0.62 a	29.76 ± 0.25 a	28.03 ± 0.22 b	29.59 ± 0.24 a
	RH	64.31 ± 1.39 a	61.12 ± 1.42 b	62.41 ± 1.44 ab	65.40 ± 0.57 a	62.44 ± 0.67 b	64.56 ± 0.69 a
2021	Air speed	0.121 ± 0.01 b	0.253 ± 0.02 a	0.143 ± 0.01 b	0.091 ± 0.00 c	0.163 ± 0.01 a	0.109 ± 0.00 b
	*T_a_*	30.98 ± 0.59 a	28.61 ± 0.58 b	30.14 ± 0.52 ab	30.23 ± 0.32 a	28.09 ± 0.26 b	29.52 ± 0.26 a
	RH	61.52 ± 1.47 a	54.90 ± 1.73 b	61.18 ± 1.52 a	62.80 ± 0.83 a	56.28 ± 0.89 b	62.05 ± 1.00 a

Note: Data in the table are means ± standard errors, and different lowercase letters after peer data indicate significant differences at the level of 0.05.

**Table 2:** Experiential parametric simulation results of the NRLD fit function for greenhouse tomato at the late growth stage under different ventilation and irrigation amounts in 2021.

Treatment	*R*_0_	*R*_1_	*R*_2_	*R*_3_	*R* ^2^	*F*
*T_R_K*_0.9_	−82.33	134.68	−70.78	12.19	0.985	19.29 **
*T_R_K*_0.5_	−97.16	152.77	−75.68	12.15	0.9621	19.88 **
*T_RS_K*_0.9_	−80.79	130.72	−67.75	11.57	0.9686	20.23 **
*T_RS_K*_0.5_	−86.21	140.18	−72.73	12.28	0.9876	20.2 **
*T_S_K*_0.9_	−79.01	128.15	−66.67	11.45	0.9775	19.94 **
*T_S_K*_0.5_	−80.80	131.13	−68.36	11.74	0.9803	20.43 **

Note: ** denotes reaching a highly significant level (*p* < 0.01).

**Table 3:** Results of NRMSE statistics for simulated and measured values of NRLD.

Treatment	*T_R_K*_0.9_	*T_R_K*_0.5_	*T_RS_K*_0.9_	*T_RS_K*_0.5_	*T_S_K*_0.9_	*T_S_K*_0.5_
2020	0.129	0.175	0.160	0.187	0.201	0.203
2021	0.193	0.273	0.159	0.195	0.209	0.220

**Table 4:** Estimating the proportion of greenhouse tomato roots allocated in 2020.

Location	Irrigation Treatment	Ventilation Treatment						Average Values	
		*T_R_*		*T_RS_*		*T_S_*			
		Simulated	Measured	Simulated	Measured	Simulated	Measured	Simulated	Measured
Z_r_ = 1/4	*K*_0.9_	73.9%	71.8%	74.3%	68.4%	74.7%	74.5%	74.3%	71.6%
	*K*_0.5_	73.4%	73.8%	74.1%	71.2%	74.3%	75.1%	73.9%	73.4%
	Average	73.7%	72.8%	74.2%	69.8%	74.5%	74.8%	74.1%	72.5%
Z_r_ = 1/2	*K*_0.9_	87.0%	86.4%	89.1%	86.1%	90.6%	87.8%	88.9%	86.8%
	*K*_0.5_	84.6%	88.4%	88.0%	83.9%	88.7%	88.3%	87.1%	86.9%
	Average	85.8%	87.4%	88.5%	85.0%	89.7%	88.1%	88.0%	86.8%

**Table 6:** RLD and yield under different treatments in 2020 and 2021.

Year	Treatment	*T_R_K*_0.9_	*T_R_K*_0.5_	*T_RS_K*_0.9_	*T_RS_K*_0.5_	*T_S_K*_0.9_	*T_S_K*_0.5_
2020	Yield (t ha^−1^)	139.5 ± 10.06 a	124.2 ± 2.47 b	147.6 ± 4.39 a	119.4 ± 7.28 b	143.1 ± 4.87 a	125.1 ± 6.53 b
	RLD (cm cm^−3^)	4.85 ± 0.72 a	4.35 ± 0.40 ab	3.82 ± 0.28 bc	3.02 ± 0.57 cd	3.15 ± 0.43 cd	2.39 ± 0.26 d
2021	Yield (t ha^−1^)	143.7 ± 9.23 ab	128.5 ± 4.65 c	148.1 ± 7.49 a	126.3 ± 4.76 c	144.5 ± 5.58 ab	130.1 ± 7.24 bc
	RLD (cm cm^−3^)	6.58 ± 1.23 a	5.22 ± 0.67 ab	3.79 ± 0.81 bc	3.27 ± 0.79 c	4.97 ± 0.58 b	2.50 ± 0.14 c

Note: Data in the table are means ± standard errors, and different lowercase letters after peer data indicate significant differences at the level of 0.05.

**Table 5:** Predicted root distribution in 2020 by setting different evaporation coefficient (1.0, 0.8 and 0.6).

Ventilation Treatment	Irrigation Treatment	Soil Depth (cm)						Zr = 1/4	Zr = 1/2
		10	20	30	40	50	60		
*T_R_*	*K*_1.0_	5.13	1.52	0.22	0.22	0.55	0.21	84.77%	90.32%
	*K*_0.8_	5.14	1.46	0.17	0.23	0.60	0.23	84.26%	89.36%
	*K*_0.6_	5.17	1.33	0.08	0.25	0.70	0.28	83.24%	87.42%
*T_RS_*	*K*_1.0_	5.11	1.63	0.29	0.20	0.46	0.17	85.63%	91.96%
	*K*_0.8_	5.12	1.57	0.25	0.21	0.51	0.19	85.18%	91.11%
	*K*_0.6_	5.13	1.52	0.21	0.22	0.55	0.21	84.72%	90.23%
*T_S_*	*K*_1.0_	5.10	1.72	0.36	0.19	0.39	0.14	86.33%	93.29%
	*K*_0.8_	5.11	1.66	0.31	0.20	0.44	0.16	85.85%	92.38%
	*K*_0.6_	5.13	1.56	0.24	0.22	0.52	0.20	85.04%	90.85%

Note: Table shows NRLD model simulation values for different soil layers (10, 20, 30, 40, 50, 60 cm), with reference to the ventilation of *T_R_*, *T_RS_* and *T_S_* in 2020, for model application.

## Data Availability

Not applicable.
